# Vegetation as a key driver of the distribution of microbial generalists that in turn shapes the overall microbial community structure in the low Arctic tundra

**DOI:** 10.1186/s40793-023-00498-6

**Published:** 2023-05-10

**Authors:** Shu-Kuan Wong, Yingshun Cui, Seong-Jun Chun, Ryo Kaneko, Shota Masumoto, Ryo Kitagawa, Akira S. Mori, An Suk Lim, Masaki Uchida

**Affiliations:** 1 Research Organization of Information and Systems, National Institute of Polar Research, 10-3, Midori-cho, Tachikawa, Tokyo Japan; 2 Division of Life Science and Plant Molecular Biology and Biotechnology Research Center, Gyeongsang National University, Jinju, 52828 Korea; 3 LMO Team, National Institute of Ecology, 1210 Geumgang-ro, Maseo-myeon, Seocheon, Republic of Korea; 4 Graduate School of Environment and Information Sciences, Yokohama National University, 79-7, Tokiwadai, Hodogaya, Yokohama, Japan; 5 Kansai Research Center, Forestry and Forest Products Research Institute, 68, Nagaikyutaroh, Momoyama, Fushimi, Kyoto Japan; 6 Department of Polar Science, School of Multidisciplinary Sciences, The Graduate University for Advanced Studies, SOKENDAI, 10-3, Midori-cho, Tachikawa, Tokyo Japan

**Keywords:** Tundra ecological network, Habitat generalist, Habitat specialist, Vascular plant, Tundra ecosystem

## Abstract

Understanding the variability of microbial niches and their interaction with abiotic and biotic factors in the Arctic can provide valuable insights into microbial adaptations to extreme environments. This study investigates the structure and diversity of soil bacterial communities obtained from sites with varying vegetation coverage and soil biogeochemical properties in the low Arctic tundra and explores how bacteria interact under different environmental parameters. Our findings reveal differences in bacterial composition and abundance among three bacterial niche breadths (specialists, common taxa, and generalists). Co-occurrence network analysis revealed Rhizobiales and Ktedonobacterales as keystone taxa that connect and support other microbes in the habitat. Low-elevation indicators, such as vascular plants and moisture content, were correlated with two out of three generalist modular hubs and were linked to a large proportion of generalists’ distribution (18%). Structural equation modeling revealed that generalists’ distribution, which influenced the remaining microbial communities, was mainly regulated by vegetation coverage as well as other abiotic and biotic factors. These results suggest that elevation-dependent environmental factors directly influence microbial community structure and module formation through the regulation of generalists’ distribution. Furthermore, the distribution of generalists was mainly affected by macroenvironment filtering, whereas the distribution of specialists was mainly affected by microenvironment filtering (species-engineered microbial niche construction). In summary, our findings highlight the strong top–down control exerted by vegetation on generalists’ distribution, which in turn shapes the overall microbial community structure in the low Arctic tundra.

**Supplementary Information:**

The online version contains supplementary material available at 10.1186/s40793-023-00498-6.

## Introduction

The Arctic region is classified as an extreme environment due to prolonged periods of wide temperature variation, freezing and thawing cycles [65], high UV radiation exposure [54], and nutrient limitations [63]. Moreover, the Arctic region is currently undergoing unprecedented disturbances as a result of global warming [33]. One such disturbance is the observed greening of the tundra ecosystem, characterized by increased plant productivity and expansion of shrubs and trees northwards and into previously treeless areas [23, 30]. Soil microorganisms, which play a crucial role in maintaining ecosystem function and soil fertility in the Arctic, are highly sensitive to climate changes and are likely to be affected by the ongoing warming of the Arctic region.

A previous study has revealed that microbial species can be categorized into different ecological groups based on their ability to adapt to various ecological niches [58]. Understanding the dimensions of these niches and the distribution of organisms can provide insights into the tolerance and responses of organisms to environmental changes [4]. Generalists, for example, are more adaptable to different environmental conditions than specialists and have a wider distribution range [76]. In contrast, specialists have narrower tolerances than generalists and restricted distributions, making them more vulnerable to extinction when environmental conditions change [70].

The distribution of microbial generalists and specialists is regulated by a combination of stochastic (neutral theory-based) and deterministic (niche theory-based) processes [43, 51]. Correlation-based microbial co-occurrence network analysis has revealed that microbial assemblages exhibit nonrandom patterns and are predominantly influenced by deterministic processes through environmental filtering [2, 27]. For example, macroenvironmental filtering focuses on the relationship between species and the macroenvironment, where species with similar traits tend to coexist and survive in the same habitat [12, 20, 38, 39, 41].

In the Arctic, the distribution of soil microorganisms is highly dependent on soil characteristics [8], vegetation types [11], and geographical distance [67]. Vegetation types influence soil biogeochemical properties [10], which in turn affect local microbiological communities in tundra soils [11, 78]. In the low Arctic, dominant bacterial communities, particularly Acidobacteria, tend to be similar regardless of vegetation type but differ in terms of relative abundance and the presence of other minor phyla [11]. In contrast, microenvironmental filtering or niche construction emphasizes on the capacity of species to modify their microenvironments through biological metabolism and individual activities and choices [12, 55]. Niche construction (microenvironment) can alter environmental filtering (macroenvironment); therefore, the use of the concept of modified environmental filtering, which includes both biotic microenvironmental filtering and abiotic macroenvironmental filtering, is recommended for more accurately predicting different interactions [29, 75].

Development of biological association networks is crucial in determining complex microbial interactions in diverse ecosystems, as they can extract new information on ecological interactions, organizational patterns, and keystone organisms and their responses to environmental variables that may not be revealed using conventional techniques [12–15, 18, 19, 22]. For instance, co-occurrence network analysis of tundra soils has revealed that a node correlated to pH is linked to the members of Alphaproteobacteria and Acidobacteria [21].

Although several studies on direct interactions between microorganisms and various environmental parameters in the Arctic have been conducted, there is still a lack of in-depth studies exploring both direct and indirect interactions and association patterns between different microbial groups that coexist in this environment. In the present study, we used network analysis and structural equation modeling to investigate the associations between different soil bacterial groups from three different elevations with distinct vegetation coverage patterns in the low Arctic tundra of Salluit. Our study aims to address the following questions: (i) which taxa belong to different niche-based ecological categories, namely, microbial generalist (broadly distributed) and specialist (restricted distribution) groups; (ii) how do these ecological categories shape the overall microbial network structure and which are the keystone species in the low Arctic; and (iii) how sensitively do microbes from different ecological categories respond to abiotic and biotic factors.

## Materials and methods

### Study area and field surveys

**Fig. 1:**
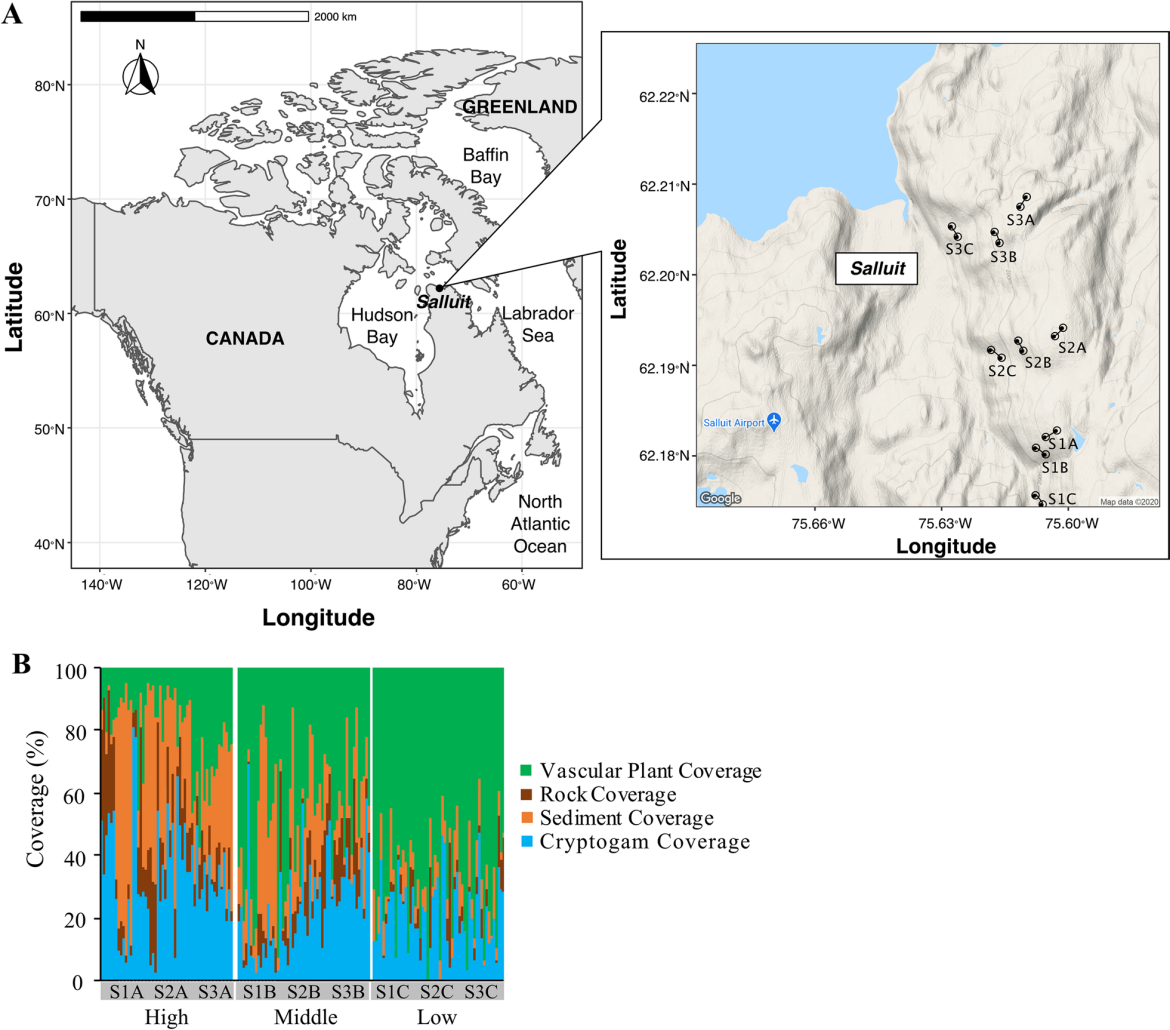
**A** Map of the study area and the nine sampling sites in Salluit, Nunavik. In total, 225 samples were collected (between the points in each sampling site). **B** Land and vegetation coverage at each sampling site

Field surveys were conducted during the summer of 2017 at Salluit, Quebec, Canada (62.1° N 75.4° W). The mean annual air temperature in Salluit is approximately − 8.5 °C, with a mean annual precipitation of 300 mm [32]. Soil sampling was conducted at three sites (S1, S2, and S3) by establishing three line transects at low-, mid-, and high-elevation areas that represented different vegetation and environmental conditions at each site (Fig. 1A. Transect A, referred to as the high-elevation area, was established on the crest of hills with limited vegetation cover; transect B, referred to as the mid-elevation area, was established in a mid-slope area with intermediate vegetation coverage; and transect C, referred to as the low-elevation area, was established in lower slopes with high vegetation coverage (Fig. 1B). Twenty-five 1 m × 1 m quadrats were set at 6 m intervals along each 150 m transect, and soil samples were randomly collected from each quadrat. In total, 225 top soil samples were collected using a sterile scoop and placed into 5-mL sterile sampling tubes containing RNA Stabilization Solution (Ambion, Austin, Texas). Samples were frozen at − 20 °C upon sampling and stored in this condition until DNA extraction.

### Soil biogeochemical analyses and vegetation cover

Soil biogeochemical analyses were conducted following previously described methods [36]. Total soil carbon (C) and nitrogen (N) concentrations were determined using an NC analyzer (Sumigraph NCH-22F, Sumika Chemical Analysis Service, Osaka, Japan). The depth of plant litter and mineral soil was measured using a 150-mm rod at three different points within each sampling quadrat. Soil samples at depths of 0.5–2.0 cm were collected after the removal of plant materials and living roots for characterization of water content (soil moisture), soil pH, electrical conductivity (EC), and C and N concentrations. EC and pH were measured using EC and pH meters, respectively (Twin EC and Twin pH, respectively; Horiba Ltd., Kyoto, Japan). Soil moisture was measured using a soil moisture sensor (HH2; DeltaT Devices Ltd., Burwell, UK), and final results were represented as the average of three readings at each sampling point. In addition, the ratio of wet and dry soil weight was estimated as a proxy for the water content of soil. Approximately 15 g of wet soil was weighed and remeasured after oven-drying at 60 °C for 48 h. The cover of vascular plant species, rock, and cryptogams (bryophytes and lichens) was recorded using a 1 m × 1 m quadrat at each sampling point. Soil temperature was measured using digital stick thermometers that were inserted into the soil to a depth of 1 cm. Measurements were performed from 10:00 to 18:00 on sunny days between July 24 and 31, 2017 to ensure consistent measurement conditions.

### Bacterial community composition analysis

DNA was extracted from approximately 0.5 g soil samples using a Fast DNA Spin Kit for Soil (MP Biomedical, Santa Ana, CA) according to the manufacturer’s instructions. The bacterial 16S rRNA gene was amplified using a universal bacterial primer set, 341F/805R (341F: 5′-CCTACGGGNGGCWGCAG-3′; 805R: 5′-GACTACHVGGGTATCTAATCC-3′), targeting the V3–V4 region of the 16S rRNA gene (Herlemann et al., 2011). PCR amplification, purification, and quantification were performed according to the Illumina 16S metagenomic sequencing library protocol. Paired-end sequencing was performed using a MiSeq Reagent Kit v3 (600 – cycle) (Illumina) according to the manufacturer’s instructions.

To investigate sequence variants of a single nucleotide in the gene, amplicon sequence variants (ASVs) of the 16S rRNA gene were calculated using DADA2 (version 1.8) following the pipeline tutorial 1.8 (date of access: March 2021; https://benjjneb.github.io/dada2/tutorial_1_8.html) in R [9]. Singletons, doubletons, and tripletons were removed from the dataset of the 16S rRNA gene to further reduce any sequencing errors produced by the MiSeq Illumina sequencing platform. The latest Silva database (release 138) was used to align and classify the sequences of the 16S rRNA gene [62]. After sequence classification, the chloroplast, mitochondria, archaea, and eukaryote sequences were removed.

To measure habitat specialization, we used the niche breadth (B) index described by Levins [42] based on the following formula:

$$B_{j} = \frac{1}{{\mathop \sum \nolimits_{i = 1}^{N} P_{ij}^{2} }},$$

where *B*_j_ and *P*_ij_ indicate niche breadth and relative abundance of species, respectively. Generalists distributed across a wider environmental range exhibit higher *B*-values than specialists. Given that extremely rare ASVs could erroneously indicate specialists, ASVs with averaged relative abundances of < 2 × 10^−5^ were excluded from network analysis and structural equation modeling (SEM) [44, 58]. Furthermore, ASVs observed in at least 25 samples (approximately 10% of the total samples) were selected for network analysis and SEM. In this study, a B-value of > 78 was chosen as a cutoff criterion for generalists, as this value lies within the outlier area of the B-value distribution [44, 45], whereas ASVs with B-values of < 22 were regarded as specialists (lower quartile) (Fig. S1), and the remaining ASVs were regarded as common taxa. The Shannon diversity index was calculated using functions in Vegan. To normalize the data for diversity indices, the reads were normalized to the lowest number of reads using the “rrarefy” function of Vegan.

### Microbial association network and topological features

To investigate the relationships among generalists, common taxa, specialists, and environmental variables, we constructed microbial networks based on microbial niche breadth, specifically the niche breadth network. This network included separate networks for generalists, common taxa, and specialists. We also constructed a total network that included generalists, common taxa, and specialists to explore the relationships between microbes and environmental variables. Spearman’s rank correlation coefficient (*ρ*) was used to construct microbial association networks, and calculations were performed using the Hmisc package [28] in R. To reduce the impact of rare ASVs in the dataset, we applied the following thresholds for each dataset: (i) at least 25 observed samples and (ii) a relative proportion of > 0.05% in at least one sample. P-values were approximated using t or F distributions, and Q-values (adjusted P-values; false discovery rate) were calculated using the Benjamini and Hochberg false discovery rate procedure [5] to account for multiple testing. For further analyses, only positive correlations with |*ρ*|> 0.5 and both P- and Q-values of < 0.001 were selected. The resulting networks were visualized using Cytoscape 3.8.2 [66], and the yFiles organic layout algorithm in Cytoscape was employed for network layout. To compare network topological features, randomly distributed Erdős–Rényi networks (with the same number of nodes and links) were used as a null model, and calculations were performed using the Network Randomizer plugin in Cytoscape. Network topological features were calculated using the “Networkanalyzer” plugin in Cytoscape and the R package igraph [1, 17]. For the purpose of comparison, Erdős–Rényi random networks were employed as null models using the Network Randomizer plugin in Cytoscape [17]. The small-world coefficient (SW) was defined as follows: SW = clustering coefficient (C/C_R_)/characteristic path length (L/L_R_), where _R_ represents parameters from a random network (Table 1). To identify the topological features of each node in the microbial network, we calculated the within-module degree (*Z*_i_) and among-module connectivity (*P*_i_) [26]. Based on their *Z*_i_ and *P*_i_ scores, nodes were classified into four roles: peripheral nodes with limited links to other nodes within their module (Zi ≤ 2.5 and Pi ≤ 0.62), connectors that are highly connected to nodes from other modules (Zi ≤ 2.5 and Pi > 0.62), module hubs that are highly linked to other nodes within their module (Zi > 2.5 and Pi ≤ 0.62), and network hubs that bridge both module hubs and connectors (Zi > 2.5 and Pi > 0.62) [57]. The modules were identified using the Louvain algorithm in the igraph package of R [6]. To calculate the distribution patterns of the major modules, the relative abundances of ASVs (except for environmental parameters) in each module were normalized using feature scaling [Xʹ = (X − X_min_)/(X_max_ − X_min_]. After normalization, the normalized ASVs in each module were averaged to obtain the distribution patterns of each module [13]. Finally, a dendrogram was constructed based on the distribution patterns of the major modules using Ward’s method, as implemented via the “hclust” function in R (version 3.4.0) (Team, 2013).

**Table 1:** Microbial association network topological features and statistics

Topological features	Generalist network (Fig. 2A)	Common taxa network (Fig. 2B)	Specialist network (Fig. 2C)	Total network (Fig. 4A)
Nodes	94	794	460	1,461
Edges	368	7112	1243	13,470
Diameter	10	12	28	13
Average number of neighbours	8.0	18.7	5.9	19.2
Network density	0.09	0.03	0.01	0.01
Network heterogeneity	0.93	1.12	0.91	1.26
Network heterogeneity, random	0.32	0.23	0.42	0.24
Centralization	0.19	0.15	0.07	0.10
Centralization, random	0.09	0.02	0.02	0.01
Modularity	0.46	0.60	0.77	0.62
Modularity, random	0.31	0.21	0.43	0.21
Average clustering coefficient (C)	0.45	0.46	0.33	0.40
Clustering coefficient, random (C_r_)	0.09	0.02	0.01	0.01
Characteristic path length (L)	4.30	4.97	9.43	5.03
Characteristic path length, random (L_r_)	2.41	2.64	3.82	2.80
C/C_r_	5.29	20.00	27.50	30.77
L/L_r_	1.78	1.89	2.47	1.80
Small-world coefficient (SW)	2.97	10.62	11.14	17.13

### GeoChip analysis

Total DNA and RNA for GeoChip analysis were extracted from 0.5 g soil samples using a ZymoBIOMICS DNA & RNA Kit (Zymo Research, Irvine, CA, USA). The total RNA extracted was subjected to DNAse I treatment, and the success of the DNAse treatment was confirmed by the absence of PCR amplification of the V1–V3 bacterial 16S rRNA gene. cDNA was synthesized from the RNA template using the SuperScript IV First-Strand Synthesis System for RT-PCR (Invitrogen, Carlsbad, CA, USA) following the manufacturer’s instructions. GeoChip 5.0 M, a comprehensive functional microarray, was used to reveal the functional diversity of the soil samples. For each soil sample, both DNA and paired cDNA samples were sent to Glomics Inc. (Oklahoma, USA) for functional gene sequencing using the GeoChip 5.0 M Microarray (Agilent Technologies Inc., Santa Clara, USA) and the method described by Shi et al. [68]. In brief, 500 ng of DNA was labeled with the fluorescent dye Cy-3 (GE Healthcare, CA, USA) using random priming with Klenow fragments, purified using a QIAquick Purification Kit (Qiagen), and then dried. The labeled DNA was suspended in hybridization solution containing 10% formamide, Hi-RPM Hybridization Buffer, aCGH blocking agent, Cot-1 DNA, and common oligonucleotide reference standards. The solution was denatured at 95 °C for 3 min, incubated at 37 °C for 30 min, loaded onto the microarray slide well, and hybridized at 67 °C for 24 h. After hybridization, the slides were rinsed and imaged with a NimbleGen MS200 microarray scanner (Roche NimbleGen, Madison, WI, USA). Functional gene names listed in this study were assigned based on the original functional gene annotations in GeoChip 5.0 M (http://ieg.ou.edu/gcs/gcsmm.cgi?version=gc50_180k). For normalization, the total abundance and expression of each category or gene were divided by those of the housekeeping gene *gyrB* [16]. Heatmaps were constructed using the function “heatmap.2” with row z-score normalization in the gplots package of R [79].

### SEM

The relationships among environmental factors, vegetation (vascular plants and cryptogams), and bacterial communities were estimated using SEM. Spearman’s rank correlation coefficient (ρ) was calculated to eliminate multicollinear environmental variables prior to SEM analysis. SEM was performed using the “sem” function in the Lavvan package [64]. The conceptual model of the hypothetical relationships proposed that (i) plant coverage was influenced by environmental factors, (ii) both plant coverage and environmental factors influenced bacterial communities (generalists, common taxa, and specialists), and (iii) generalists drove the dynamics of common taxa and specialists, with common taxa further influencing specialists. Plant coverage was considered a latent variable indicated by vascular plant and cryptogam coverage. The first detrended correspondence analysis (DCA) axis scores were used in the subsequent SEM analysis for generalists, common taxa, and specialists.

### Statistical analyses

All statistical analyses were performed using the R package (version 3.4.0) [73]. To compare the differences in the compositions of generalists, common taxa, and specialists among elevations, we used DCA with the “decorana” function in Vegan [56]. Differences in taxonomic composition among elevations were tested using PERMANOVA with 999 permutations, employing the “adonis” function in Vegan. The relationships between environmental parameters and bacterial community composition were examined by fitting vectors onto the ordination space using the “envfit” function in Vegan. The significance of the fitted vectors was assessed using a permutation procedure with 999 permutations. One-way analysis of variance followed by Tukey’s honest significant difference test were used to determine significant differences in environmental parameters among the three elevations.

## Results

### Environmental parameters

In total, 225 soil samples were categorized into three groups based on sampling elevation and vegetation coverage: high (crest, low vegetation coverage), middle (mid-slopes, intermediate vegetation coverage), and low (lower slopes, high vegetation coverage) at 302 ± 39, 251 ± 21, and 162 ± 43 m, respectively (Fig. 1A). Environmental parameters and vegetation coverage varied significantly among the elevations (Additional file 1: Table S1; Fig. 1B). For example, vascular plant coverage in low-elevation samples was approximately four times higher (70%) than that in high-elevation samples, whereas cryptogam coverage was significantly higher in high-elevation samples (Table 1; Fig. 1B). Top soil temperature increased significantly with elevation, ranging from 10.2 to 17.1 °C. Moisture content ranged from 18 to 59%, with the highest and lowest values observed at lower and higher elevations, respectively (Additional file 1: Table S1). The average C/N ratio was 13.1 ± 2.6, with significantly higher values at low elevations (Additional file 1: Table S1). The mean pH value across the sampling areas was 5.1 ± 0.3, and no significant differences in pH values were observed among the three elevations (Additional file 1: Table S1).

### Bacterial community composition and habitat specialization

**Fig. 2:**
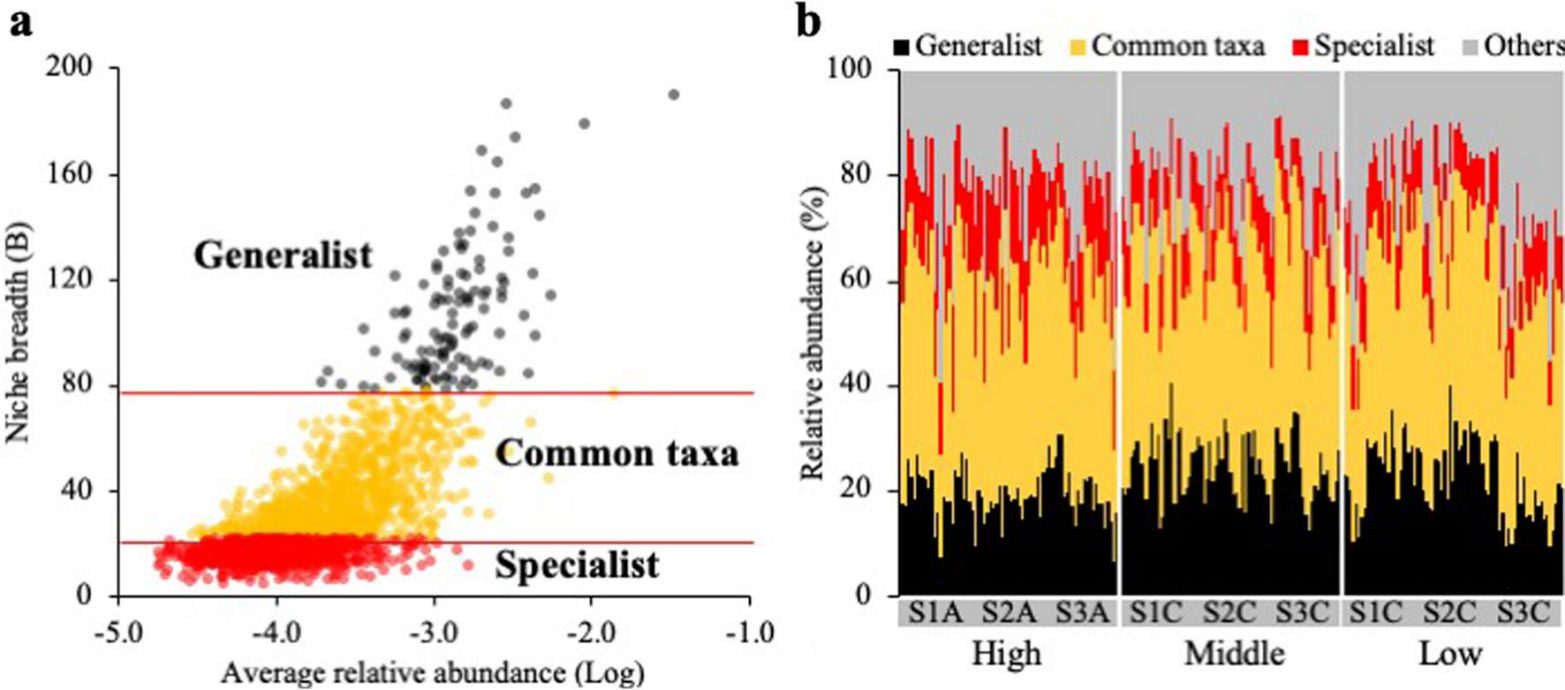
**A** Niche breadth (B) of the specialists, common taxa, and generalists. The x-axis indicates average relative log-abundances. Each dot represents an individual amplicon sequence variant (ASV). The ASVs of generalists (B > 78), common taxa (22 ≤ B ≤ 78), and specialists (B < 22) are indicated in black, red, and orange, respectively. **B** Relative abundance of generalists, common taxa, and specialists in the total bacterial community composition

In total, 19,979 ASVs were identified from 7,699,439 high-quality reads in 225 soil samples, with an average of 835 ± 503 ASVs in each sample. Among all sampling sites, the phylum Proteobacteria (27.9%) was the most abundant microbial group, followed by Acidobacteria (19.5%), Verrucomicrobiota (12.9%), Chloroflexi (11.1%), and Actinobacteriota (9.7%). After removing rare ASVs (refer to the Materials and Methods for the exclusion criteria), 2,141 ASVs remained for further analysis based on the niche breadth index (Fig. 2A). Among these, 112 (22.2% ± 6.4%), 1190 (41.0% ± 6.3%), and 839 (10.0% ± 3.2%) ASVs were identified in generalists, common taxa, and specialists, respectively (Fig. 2B).

**Fig. 3:**
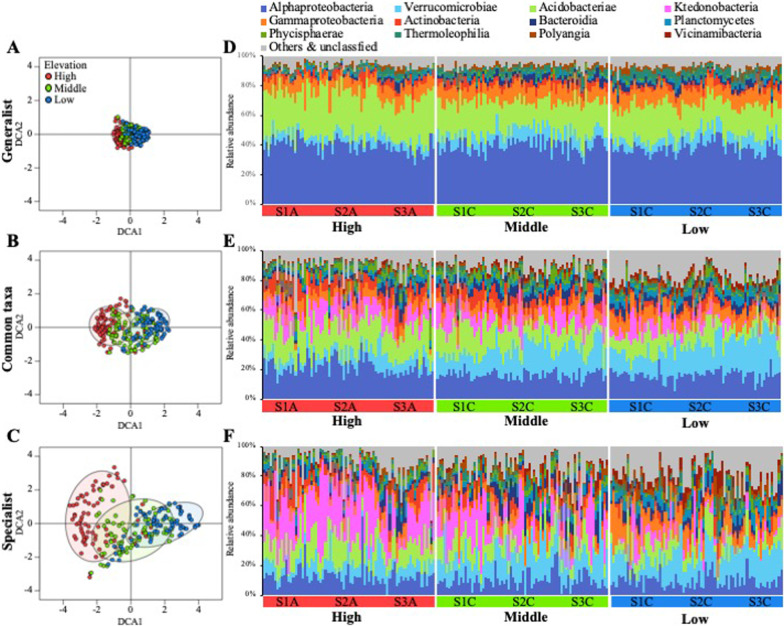
Detrended component analysis (DCA) plots based on the similarity of ASV composition in **A** generalists, **B** common taxa, and **C** specialists. The relative abundance of **D** generalists, **E** common taxa, and (F) specialists at the class level

To explore the prevailing patterns of generalist, common taxa, and specialist compositions and identify environmental parameters serving as potential drivers, we performed DCA. Microbial variation was more significant among specialists than among common taxa or generalists (Fig. 3A–C) and was segregated according to low-, mid-, and high-elevation levels (PERMANOVA; generalists, common taxa, and specialists pseudo‐*F* = 5.21, 3.93, and 2.46, respectively; *p* < 0.001). The microbial variation for the three niche-based groups was significantly influenced by elevation level, vascular plant coverage, soil temperature, and C/N ratio (Additional file 1: Table S2). However, no significant difference was observed in the bacterial diversity index among the sampling sites (Additional file 1: Fig. S2).

At the class level, major microbes among generalists, common taxa, and specialists displayed significant differences in their abundances (Fig. 3D–F). For instance, the proportions of Alphaproteobacteria and Acidobacteriae were two- to three-fold higher among generalists than among specialists (t-test, *p* < 0.001), whereas Ktedonobacteria and Vicinamibacteria were absent among generalists. In contrast, the proportions of Verrucomicrobiae, Planctomycetes, Phycisphaerae, and Bacteroidia were two- to three-fold higher among specialists than among generalists (t-test, *p* < 0.001). As expected, the microbial components were relatively evenly distributed in the generalists’ group compared with the specialists’ group.

### Niche breadth-based distribution networks and modules

**Fig. 4:**
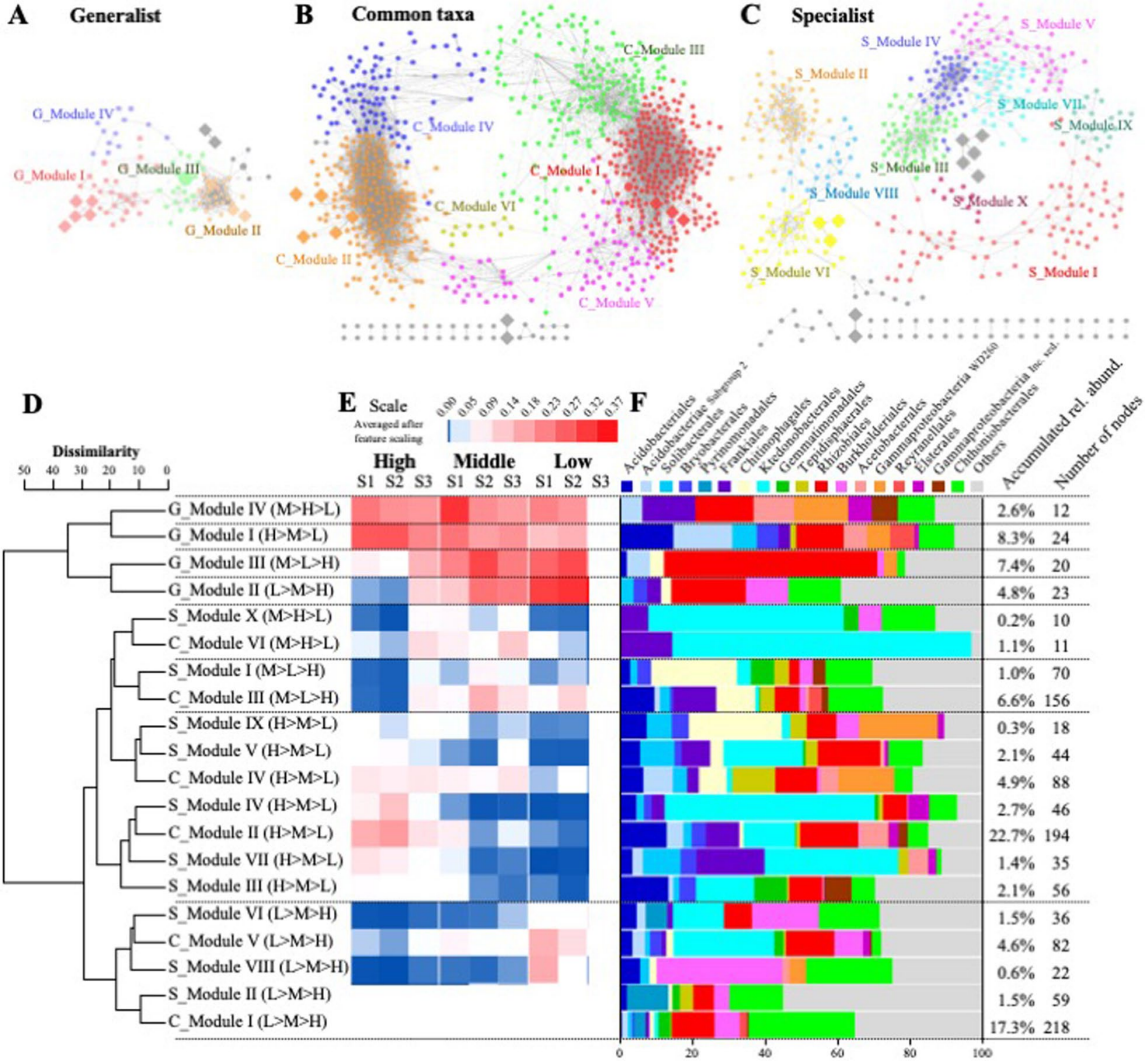
Specific microbial association networks with **A** generalists, **B** common taxa, and **C** specialists. Node colors represent major modules. **D** Dendrogram and heatmap obtained via hierarchical clustering analysis based on the distribution patterns of individual modules. **E** The average relative abundance of bacterial groups in the major modules at the order level

Niche breadth-based distribution networks were constructed using the ASVs assigned to the generalists, common taxa, and specialists along with 15 different environmental parameters. These networks were designated as generalist, common taxa, and specialist networks (Fig. 4A–C), and their topological features are summarized in Table 1. The characteristic path length, representing the average shortest path between all pairs of species, was approximately two-fold higher in the specialist network than in the common taxa and generalist networks. Network centralization was lower in the specialist network (0.07) than in the common taxa (0.15) and generalist (0.19) networks. However, the specialist network exhibited the highest modularity (0.77), and the modularity of each niche breadth-based distribution network was higher than that of its Erdős–Rényi random network. In addition, all SW values, with higher values indicating higher small-worldliness of the network, were > 1, suggesting that all niche breadth-based distribution networks in this study exhibited small‐world properties.

Each niche breadth-based distribution network comprised 4–10 major modules, which occupied 87%–95% of the total nodes in each association network. Dendrograms constructed using the major module distribution patterns allowed separation of the major modules into several different groups related to the elevation types of samples (Fig. 4D). As expected, the modules of generalists (G_Modules) were clustered into a single group, displaying relatively less variation among elevations. However, the module patterns of common taxa (C_Modules) and specialists (S_Modules) were elevation-dependent. For instance, C_Modules I and V and S_Modules II, VI, and VIII were clustered together, and their proportions were higher at low elevations, whereas the proportions of C_Modules II and IV and S_Modules III, IV, V, VII, and IX were higher at high elevations. The major bacterial components of the modules varied significantly for each of the individual modules but were generally similar across closely clustered modules in the dendrogram (Fig. 4E). For instance, Rhizobiales was the major group in the G_Modules, whereas Ktedonobacterales and Chitinophagales were abundant in the C_Modules and S_Modules.

### Total network and modules

**Fig. 5:**
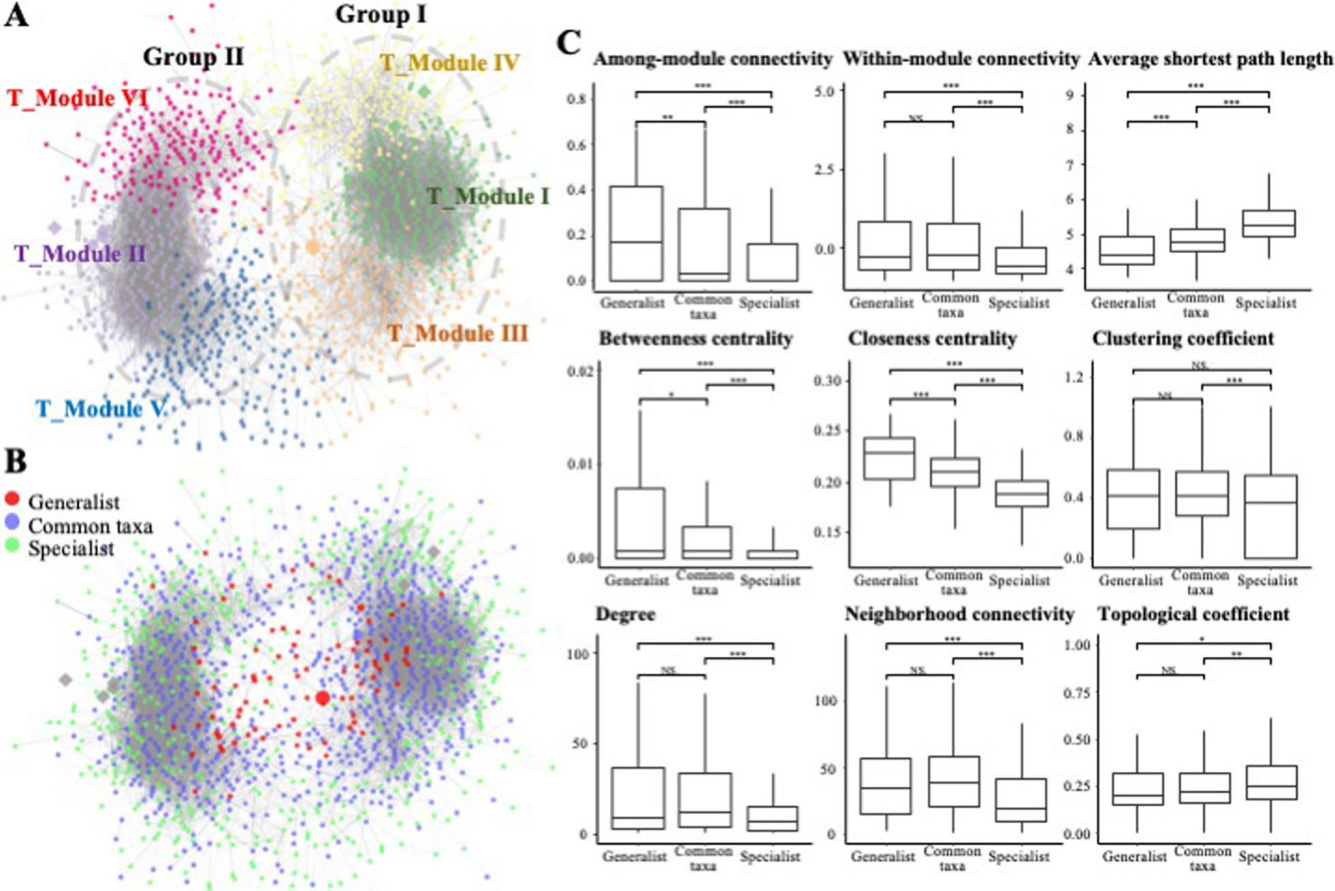
Total microbial association network representing **A** major modules and **B** generalist, common taxa, and specialist ASVs. **C** Topological characteristics of generalist, common taxa, and specialist ASVs in the total microbial association network. *: 0.01 ≤ *p* < 0.05; **: 0.001 ≤ *p* < 0.01; ***: *p* < 0.001

The total network (T_Module) comprised six major modules, which occupied 96% of the nodes in the network (Fig. 5A). In contrast to the niche breadth networks, the structure of the total network was topologically similar to that of the common taxa network (Fig. 4B). For instance, approximately 98% of the common taxa ASVs in T_Modules I, II, III, and V were derived from C_Modules I, II, III, and IV, respectively (Additional file 1: Fig S3). The topological parameters of the total network were also similar to those of the common taxa network (Table 1). T_Modules I and II were located at the center of Group I and Group II and were connected to other modules on both sides. Specifically, the ASVs of generalists and common taxa were located at the center of the network and the center of each module, respectively, whereas those of specialists were located on the sides of the network (Fig. 5B). The topological characteristics of the ASVs of generalists, common taxa, and specialists in the total network also supported these results (Fig. 5C). The among-module connectivity, betweenness centrality, and closeness centrality values were significantly higher in the generalist group than in the common taxa and specialist groups. In contrast, compared with the generalist and common taxa groups, the specialist group showed a high average shortest path length value as well as low within-module degree and neighborhood connectivity values.

The relative abundance of generalists in T_modules I and II was approximately two-fold higher than that in other modules (Additional file 1: Fig. S3B). However, the relative abundance of common taxa was two-fold higher in T_modules III and V than in other modules (Additional file 1: Fig. S3B). The bacterial community structure of the total network also differed among modules (Additional file 1: Fig. S3B). For instance, Rhizobiales, the most abundant group of generalists, appeared to be dominant in most modules; however, Ktedonobacterales, the most abundant group of specialists, was also dominant as a specialist group in T_module II. The distribution patterns of each T_module were calculated and linked to different environmental variables (Additional file 1: Fig. S4). Altitude, respiration, moisture, dry/wet ratio, and vascular plant coverage were the main environmental variables that correlated with the distribution patterns of individual modules. T_Modules I and II showed opposite distribution patterns, with T_Module I being positively correlated with moisture and vascular plant coverage and T_Module II being positively correlated with altitude, respiration, and soil dry/wet weight ratio. T_Module I, but not T_Module IV, was weakly positively correlated with temperature. In contrast, T_Module IV, but not T_Module I, was weakly positively correlated with C/N ratio. Therefore, although T_Modules I and IV showed similar distribution patterns and were closely clustered, their correlation patterns in relation to temperature and C/N ratio were different.

**Fig. 6:**
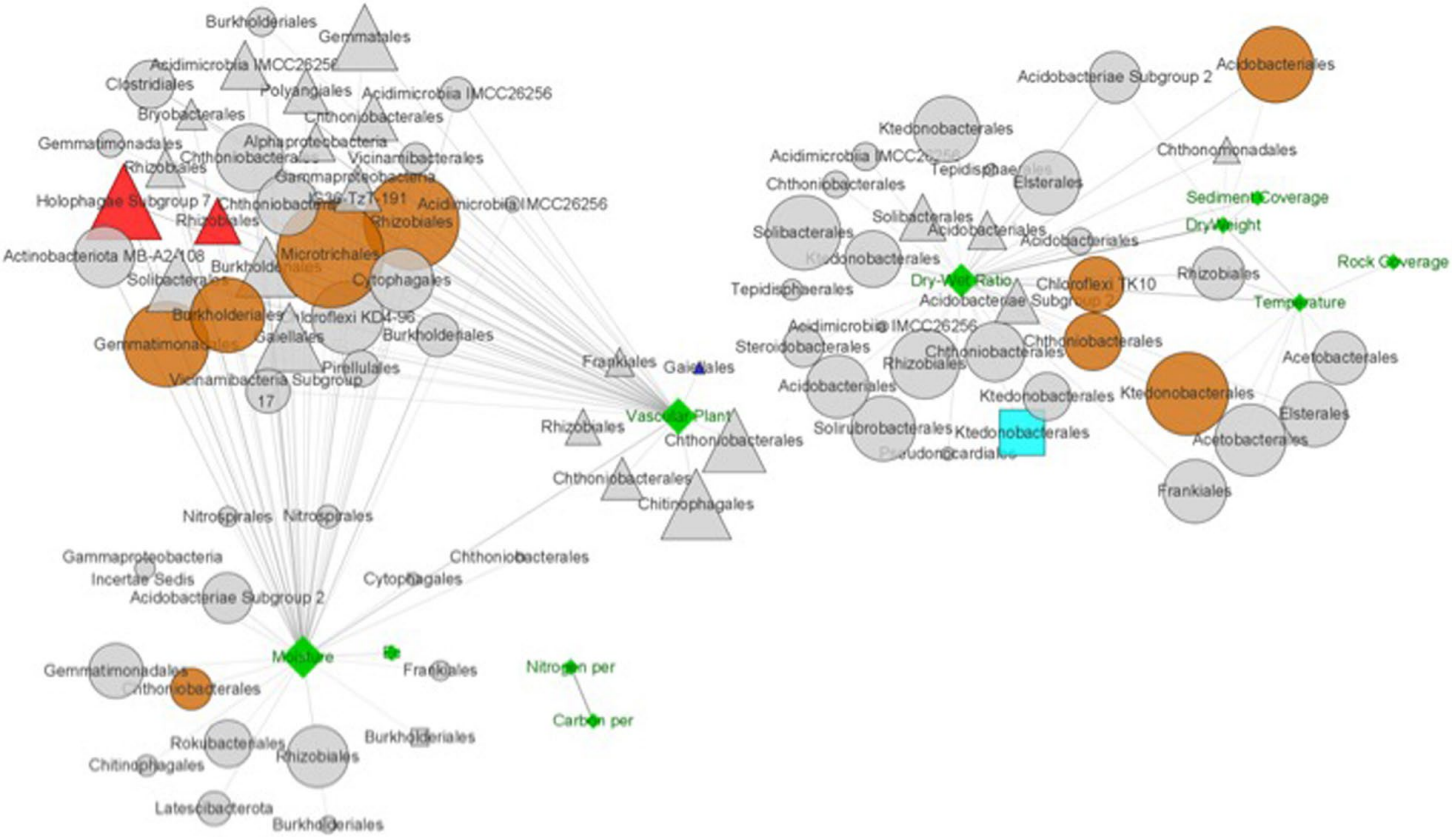
Subnetwork constructed using environmental factors and their directly linked microbes. Triangle, generalists; circle, common taxa; rectangle, specialists; diamond, environmental factors. Module hubs from generalists, common taxa, and specialists are colored red, brown, and cyan, respectively, whereas environmental factors are colored green

To investigate the links between environmental factors and individual microbial taxa, we constructed a subnetwork consisting of 10 different environmental factors and nodes containing the microbial taxa to which they were directly linked (Fig. 6). Interestingly, the majority of nodes that were linked to vascular plant coverage (51%) and moisture content (28%) were from habitat generalists. Considering the number of generalists in this study, 21% and 14% of generalists were correlated with vascular plant coverage and moisture content, respectively. However, temperature and soil dry/wet weight ratio were closely linked to common taxa; the proportions of common taxa within all nodes that were linked to temperature, soil dry weight, and soil dry/wet weight ratio were 100%, 75%, and 84% respectively. Two correlations were observed between environmental factors and microbes: one with moisture content and the other with soil dry/wet weight ratio. Approximately 30–40% of microbes that were correlated with vascular plant coverage and moisture content were assigned to Burkholderiales, Chthoniobacterales, and Rhizobiales, whereas approximately 40–50% of microbes that were correlated with soil temperature and soil dry/wet weight ratio were assigned to Acidobacteriales, Chthoniobacterales, and Ktedonobacterales.

### Module hubs and connectors in the total network

To assess the importance of individual nodes in the tundra ecosystem, we calculated *Z*_i_ and *P*_i_. The *Z*_i_ score indicates how well the node *i* is connected to other nodes within its own module, whereas the *P*_i_ score indicates how well the node *i* interacts and connects with nodes from other modules, forming a more coherent network. In short, *P*_i_ = 0 if the node is only linked to nodes within its own module, whereas *P*_i_ → 1 if the node is evenly linked to other nodes from other modules within the network.

Out of 1,461 nodes detected in this study, we observed 42 module hubs (*Z*_i_ score > 2.5), which accounted for 2.9% of the total nodes, and 6 connectors (C score > 0.62), which occupied < 0.5% of the total nodes. Among the 42 modular hubs, 3, 36, and 3 were from generalists, common taxa, and specialists, respectively. Three connectors each were observed in generalists and common taxa (Additional file 1: Table S3). Approximately 20% of total edges were related to the aforementioned module hubs.

Taxonomically, the module hubs and connectors consisted of a wide variety of bacterial groups. The top four most observed bacterial groups at the order level were Rhizobiales (nine module hubs and one connector), Ktedonobacterales (six module hubs and two connectors), Chthoniobacterales (three module hubs), and Tepidisphaerales (three module hubs). Notably, vascular plant coverage and moisture content (indicators of low-elevation areas) were correlated with six and seven module hubs, respectively, of which two belonged to generalists (Fig. 6). Considering that only three module hubs in the network were from generalists, these two environmental factors were correlated with two-thirds of the module hubs of generalists. In addition, the soil dry/wet weight ratio was correlated with five module hubs, including four and one from common taxa and specialists, respectively. Only three module hubs in the network were from specialists; therefore, the soil dry/wet weight ratio was related to one-third of the module hubs of specialists.

### Comparison of vegetation-related ecological functional structures

**Fig. 7:**
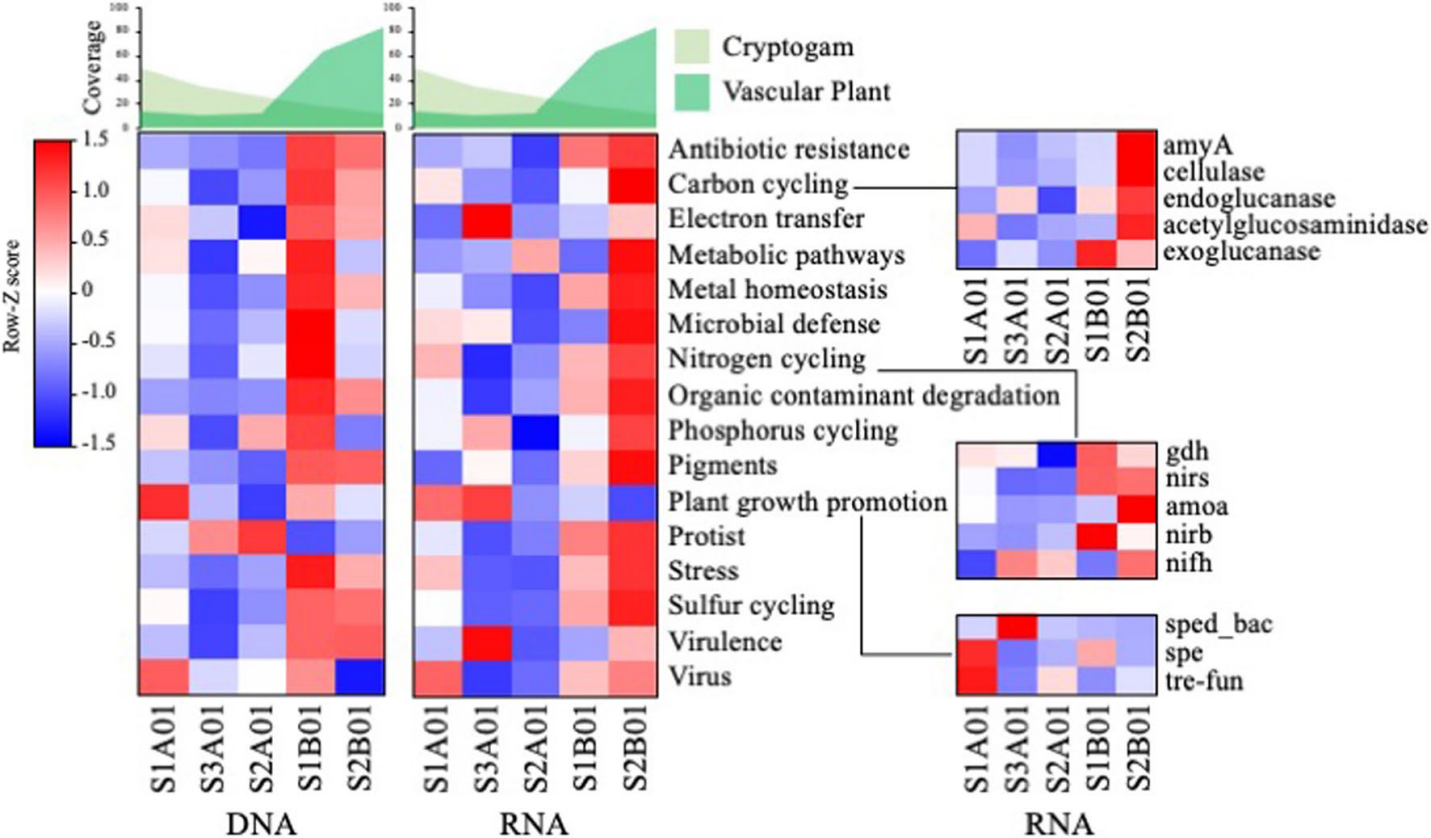
Heatmap representing the abundance (Z-scores) of gene categories detected using Geochip 5.0 M at both DNA and RNA levels

In total, 1185 genes belonging to 16 functional categories, including virulence (389), carbon cycling (132), metal homeostasis (115), stress (102), virus (101), organic contaminant degradation (100), microbial defense (63), and nitrogen cycling (26), were detected using GeoChip 5.0 M. With the exception of functions related to the categories plant growth promotion, protist, and virus, most functions exhibited higher values in vascular plant-dominant samples (S1B01 and S2B01) at the DNA level (Fig. 7). Gene expression (RNA) related to C and N cycling was higher in vascular plant-dominant samples (S1B01 and S2B01), whereas that of genes related to plant growth promotion was higher in cryptogam-dominant samples (S1A01 and S3A01). In contrast, gene expression related to cellulose degradation, such as cellulase, endoglucanase, and exoglucanase gene expression, increased in vascular plant-dominant samples.

### SEM

**Fig. 8:**
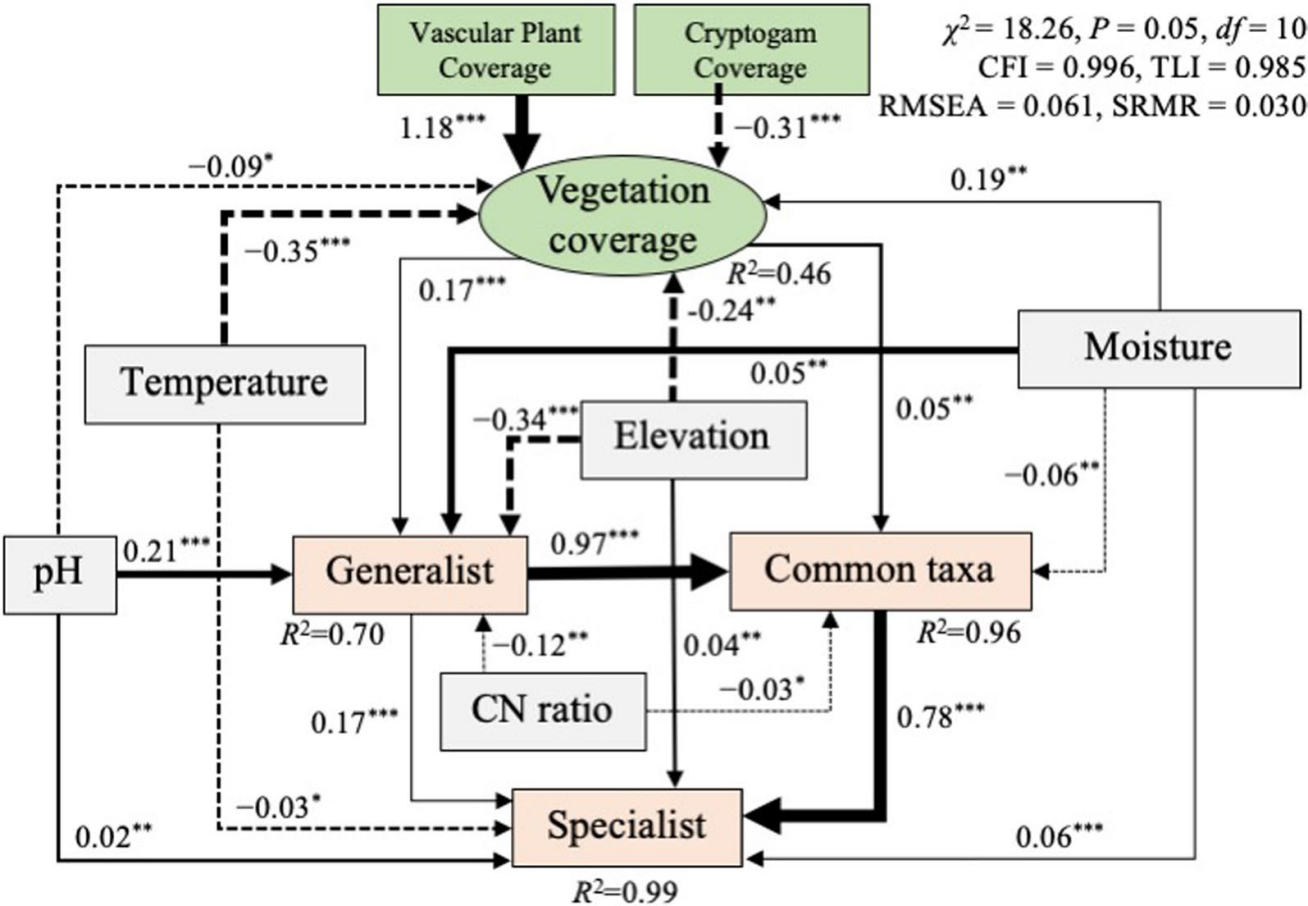
Structural equation model (SEM) representing the relationships among environmental factors, plant coverage, generalists, common taxa, and specialists. The solid and dotted arrows indicate positive and negative effects, respectively. The width of the arrows indicates the strength of the effect. ^***^: *p* < 0.001; ^**^: *p* < 0.01; ^*^: *p* < 0.05

A hypothetical model linking different environmental variables; vegetation coverage; and the dynamics of generalists, common taxa, and specialists was constructed and evaluated (Fig. 8). Several candidate models were compared, and the model that best represented the data was selected. The final structural equation model exhibited the following values: χ^2^ test statistic = 18.26 with 10 degrees of freedom; RMSEA = 0.061; CFI = 0.996; TLI = 0.985; SRMR = 0.030 (Fig. 8). These fit indices indicated that the model remained within acceptable limits and had a good fit for its purpose. In addition, the SEM results revealed that temperature, elevation, and moisture content were the major abiotic environmental factors influencing vegetation coverage. Vegetation coverage, along with pH, moisture content, and elevation, affected the dynamics of generalists. Common taxa were mainly affected by generalists and partly by vegetation coverage and moisture content. Specialists were mainly affected by common taxa and partly by generalists, pH, temperature, elevation, and moisture content, although they were not directly affected by vegetation coverage. Overall, the hypothesized models explained 46%, 70%, 96%, and 99% of the variance in vegetation coverage, generalists, common taxa, and specialists, respectively (Fig. 8).

## Discussion

The presence of higher trees and their diversity and richness play pivotal roles in maintaining a stable soil microbiome in natural ecosystems [69, 74, 77]. However, in contrast to temperate regions, the tundra ecosystem is characterized by harsher environmental conditions due to the absence or scarcity of higher trees, resulting in lower biotic diversity and simple vegetation structures. Vascular plants and cryptogams, which survive under the snow during the cold season, are the most commonly observed plant types in the tundra ecosystem [52]. As a result, the distribution patterns of vegetation and their interactions with microbes are deterministic factors that influence soil biodiversity in the tundra ecosystem [81]. For example, the coverage level of cryptogams has been shown to affect the structure of bacterial and fungal communities and play a crucial role in the establishment of vascular vegetation in Arctic soils [35]. Furthermore, vegetation patches increase microbial activity by providing bioavailable substances [61]. Our results revealed that in the tundra, vascular plants were dominant in low-elevation areas, whereas cryptograms, particularly bryophytes and lichens, were dominant in high-elevation areas (Fig. 1B). These vegetation distribution patterns in low-elevation areas could be attributed to lower environmental stresses, higher species competition, and higher resource availability compared with those at higher elevations [36].

Microbial distribution patterns and their functions also varied in relation to environmental factors and vegetation dynamics. The distribution of habitat generalists was relatively uniform, whereas the distribution of habitat specialists varied more significantly compared with that of common taxa and generalists (Fig. 3), suggesting that macroenvironment and microenvironment filtering play important roles in controlling the distribution of microbes in specific ecological niches. Network analysis showed that Rhizobiales, Ktedonobacterales, and Chthoniobacterales served as keystone microbes (either as module hubs or connectors) in the total network, indicating their essential roles in the tundra ecosystem. SEM revealed that vegetation coverage directly regulated the microbial structure of generalists and indirectly affected the microbial structures of common taxa and specialists (Fig. 8). Furthermore, microbial regulation acting on specialists was found to be greater than regulation by abiotic factors, suggesting that microbial niche construction (or microenvironment filtering) is essential for the survival of specialists in the tundra ecosystem. In summary, the vegetation level in tundra ecosystems exerts top–down control on habitat generalists, which in turn affects the overall microbial community composition and microbial modular structure formation. Thus, species-engineered microbial niche construction, rather than environmental fluctuations or macroenvironmental filtering, appears to be the fundamental factor controlling the communities of habitat specialists.

Our results revealed that niche breadth was associated with elevation-dependent unique microbial communities, with higher variation observed in specialists (Fig. 3). Specialists, with restricted niche breadth and highest fitness in their optimal habitat, are more susceptible to subtle changes in the environment than generalists [37]. Given that we removed rare ASVs observed in < 25 samples, which were mostly assigned to specialists, this would theoretically make the specialists’ network more complex. Consistent with previous results in marine [50] and terrestrial soil [3] ecosystems, the specialists in the present study showed a higher modularity value and formed more modules than the common taxa and generalists in the niche breadth network, with variation patterns exhibiting elevation-dependency (Fig. 4C). It has been reported that microbial module formation is driven by both macroenvironment filtering and species-engineered microenvironment filtering [12]. Therefore, high interspecies interactions among specialists could be a key mechanism underlying adaptation to specific conditions and survival under the influence of fluctuating environment conditions. Collectively, these findings suggest that specialists formed an independent microbial cluster to produce a specific microenvironment, i.e., niche construction, which could help them persist and avoid extinction in the harsh tundra ecosystem.

Environmental disturbances and habitat heterogeneity are known to be advantageous for the succession of generalists rather than specialists [24, 49]. Therefore, determining the mechanisms driving the coexistence of generalists and specialists in ecosystems remains a central challenge in ecology [71, 80]. However, the sensitivity of generalists and specialists to environmental fluctuations and their mutual influence have not been thoroughly explored [53]. Several studies have reported that biodiversity is primarily driven by common taxa that are sensitive to environmental changes [31, 34, 71]. Consistent with previous studies, the overall structure of the total network was similar to that of the common taxa network. As expected, the common taxa occupied approximately 60% of the total nodes, were the main component in module hubs (36 ASVs), and comprised half of the connectors (3 ASVs) in the total network. Therefore, we assumed that the common taxa played a major role in the ecological network in the tundra ecosystem. In contrast, the generalists occupied only 7% and 50% of the total module hubs and connectors, respectively. Considering that the generalists accounted for only approximately one-tenth of the nodes of the common taxa, approximately 7% of the generalists acted either as module hubs or connectors, proportionally exceeding those in the common taxa (5%). Furthermore, the overall values of nodes assigned to the generalists showed high among-module connectivity and betweenness centrality as well as high within-module degree and node degree. These results suggest that the generalists, despite having small number of ASVs, can act as bridges that connect and tie the microbial communities together and function as hub species that assist other microbes with their essential substances [12, 18, 26]. In contrast, the specialists showed the lowest values for the aforementioned indices, indicating that they interacted actively within their own group and with common taxa but less with generalists. Based on these findings and the results observed in the niche breadth-based distribution network discussed earlier, we assume that specialists, which have restricted niche breadth and high fitness under local conditions relative to generalists [37], preferably make selective connections with others within the same niche to form species-engineered microenvironments that allow them to persist and thrive in harsh tundra environments.

In ecological networks, module hubs play crucial roles as keystone taxa, assisting other microbes and maintaining the structure and function of the network [22]. The loss of connectors and module hubs can lead to the collapse and deterioration of the entire ecological network [26]. Our study revealed that Rhizobiales and Ktedonobacterales acted as module hubs and/or connectors, comprising 36% of the total module hubs and 50% of the connectors in the total network. These two groups, apart from being keystone microbes, were found to be the most dominant groups in both generalists (Rhizobiales) and specialists (Ktedonobacterales), highlighting their essential roles in the study sites. Previous studies have reported that Rhizobiales and Acidobacteriales are dominant bacterial groups in the Arctic region [45, 46]. The average relative abundance of Rhizobiales in our study was 12.2% ± 2.3%, which is approximately five-fold higher than that in unplanted soil environments and similar to that in plant microbiota (5–17%) [25]. The presence of Rhizobiales as a keystone species indicates that plant root activities influence microbial co-occurrence relationships in the soil. As the predominant module hub group, Rhizobiales was connected to a wide variety of microbial groups, such as Microtrichales and Cytophagales, in the tundra microbial network (Table 2, Fig. 5). These three groups have been reported to be involved in the degradation process of plant residues and can affect soil organic carbon content [40, 72]. Vascular plant coverage and moisture content, two important environmental factors, were directly correlated with module hub of Rhizobiales in generalists. Given that Rhizobiales is one of the most abundant bacterial groups across various environments and a core member of plant microbiota, including plant symbionts with nitrogen-fixing abilities [25], it is likely that Rhizobiales interact symbiotically with plants and support other microbes through molecular and genetic information transfer [7]. Therefore, Rhizobiales and their ecologically associated microbial neighbors are essential for the growth of tundra plants, and in turn, local plants may help generalists survive and thrive in the tundra ecosystem.

**Table 2:** Number of edges among the modules

	Group I			Group II		
	Module I	Module III	Module IV	Module II	Module V	Module VI
Module I (n = 283)	4194 (10.5%)	305 (0.4%)	336 (0.6%)	–	–	1 (0%)
Module III (n = 257)	305 (0.4%)	1073 (3.3%)	19 (0%)	2 (0%)	38 (0.1%)	4 (0%)
Module IV (n = 203)	336 (0.6%)	19 (0%)	644 (3.1%)	2 (0%)	1 (0%)	24 (0.1%)
Module II (n = 268)	–	2 (0%)	2 (0%)	4200 (11.7%)	365 (0.7%)	449 (0.9%)
Module V (n = 196)	–	38 (0.1%)	1 (0%)	365 (0.7%)	794 (4.2%)	5 (0%)
Module VI (n = 181)	1 (0%)	4 (0%)	24 (0.1%)	449 (0.9%)	5 (0%)	846 (5.2%)

Ktedonobacterales was identified as one of the dominant specialist groups in samples collected from high-elevation areas. The predominant ASV in Ktedonobacterales (ASV0005) showed high 16S rRNA gene similarities (> 98%) with uncultured bacterium clones obtained from the alpine tundra, Mount Mila in the Tibetan Plateau, and Mount Grand Galibier in the Cottian Alps [59, 83]. This finding suggests that this group is well-adapted to Arctic and alpine tundra ecosystems and is commonly found in global tundra soil ecosystems. Ktedonobacteria is characterized by large genome size and a high ratio of hypothetical proteins with unknown functions, although functions related to plant biomass degradation through strong cellulolytic activity have been reported [82]. Despite the low level of primary productivity, the active soil layer in permafrost contains significant amounts of organic matter [60], providing a favorable environment for multifunctional microbial groups, such as Ktedonobacteria, to adapt and thrive in this area. Approximately 75% of ASVs assigned to Ktedonobacteria were observed in T_Module II, T_Module V, and T_Module VI, and the distribution patterns showed positive correlations with altitude. No correlations were observed between ASVs assigned to Ktedonobacterales and vascular plant coverage or moisture content, but Ktedonobacterales showed correlations with soil dry/wet weight ratio and soil temperature, either as module hubs or high-degree nodes. As mentioned earlier, Ktedonobacterales contained the second most abundant module hubs in the present study, which could be attributed to their multifunctional genomic features [82]. Being a module hub, this microbial group requires the ability to undertake multiple biogeochemical processes to provide other microbes with indispensable substances [26]. The large genome size and high ratio of functionally unknown hypothetical proteins [82] may give Ktedonobacteria a genetic advantage that allows them to perform multiple functions in complex biogeochemical processes.

Environment filtering is a critical factor that influences shifts in microbial communities [38, 55]. Numerous studies have shown that environmental factors are the primary drivers of microbial community structures in diverse habitats. Both the niche breadth-based distribution networks and total network were divided into several major modules, some of which reflected the distribution patterns of specific environmental factors, including vascular plant coverage, moisture content, and soil dry/wet weight ratio (Additional file 1: Fig. S4). This finding is consistent with that of previous studies in aquatic and terrestrial ecosystems [12, 13, 18, 47, 48]. Our model revealed that vegetation coverage, along with other abiotic environmental factors, was the major driver of generalists, and to some extent, directly influenced common taxa. In contrast, specialists were not directly influenced by these biotic and abiotic factors (Fig. 8). In the total network, almost all ASVs that correlated with vascular plant coverage and moisture content were from generalists or common taxa, with an average of 18% of generalists being correlated with these two environmental factors. Notably, one-third of module hubs derived from generalists were directly correlated with the two environmental factors mentioned above. Given that generalists and common taxa constituted an average of > 60% of the total microbial communities, we assumed that these microbial communities were affected by vegetation coverage. Therefore, macroenvironment filtering could play a crucial role in shaping the variation in overall microbial community structures, particularly in the case of habitat generalists. Compared with generalists, specialists appeared to be less affected by vegetation coverage, as species-engineered microbial niche construction (or microenvironment filtering) was the key factor in shaping the variation of habitat specialists in the tundra ecosystem. In summary, tundra vegetation coverage, which is influenced by abiotic environment factors, controls overall microbial community structure and module formation by directly regulating habitat generalists.

## Conclusion

The present study highlights the relationships between microbial niche breadth and vegetation patterns in the tundra ecosystem. Different niche breadths were associated with different microbial communities, with habitat generalists being largely influenced by macroenvironment filtering effects and habitat specialists being primarily affected by microenvironment filtering. Notably, Rhizobiales and Ktedonobacterales served as keystone microbes, playing critical roles in supporting other microbes in the tundra ecosystem through key metabolic functions. Furthermore, vegetation coverage directly regulated the distribution of the microbial communities of generalists, which in turn affected the distribution of the other microbial communities within the ecosystem. In summary, tundra vegetation coverage exerted top–down control on habitat generalists, which in turn influenced the composition of the remaining microbial communities and the formation of microbial modular structures. Importantly, species-engineered microbial niche construction, rather than the environmental fluctuations, emerged as the fundamental factor affecting the communities of habitat specialists.

## Supplementary Information

**Additional file 1: Table S1.** Results of one-way ANOVA and post-hoc Tukey’s HSD testing among-elevation variation in environmental and biotic variables. For the Tukey HSD column, elevation not sharing a letter are significantly different. **Table S2. **Environmental fitness of environmental parameters correlated with DCA ordinations of Generalists, Common taxa, and Specialists. **Table S3.** Module hubs and connectors in Total network and their topological characteristics and taxonomical features. **Figure S1. **The distribution of the niche breadthvalues of the ASVs. A B-value of >78 was chosen as a criterion for generalists as this value lies within the outlier area of the B-value distribution, while ASVs with B-values of <22 were regarded as specialists. **Figure S2 **Shannon diversity index of the nine different sampling sites in Salluit, Nunavik. **Figure S3.**The relative abundance of Generalist, Common taxa, and Specialist modules in the total network module.Composition of major modules. **Figure S4.** Spearman's rank correlation matrix between the variables including the distribution patterns of individual modules and environmental parameters. The colors of the scale bar represent Spearman's correlation coefficient

## Data Availability

The raw amplicon sequences and accompanying metadata have been deposited in the National Center for Biotechnology Information Sequence Read Archive under the project number PRJNA713293. All raw and processed GeoChip 5.0 M data were deposited to NCBI Gene Expression Omnibus database (GEO) under the accession number GSE168623.
