# Supplementary material for: Vegetation as a key driver of the distribution of microbial generalists that in turn shapes the overall microbial community structure in the low Arctic tundra
Source: Environ Microbiome. 2023 May 10;18:41. doi: 10.1186/s40793-023-00498-6 (PMC10173506; doi:10.1186/s40793-023-00498-6)
Supplement: Supplementary file 1 — Additional file 1: Table S1. Results of one-way ANOVA and post-hoc Tukey’s HSD testing among-elevation variation in environmental and biotic variables. For the Tukey HSD column, elevation not sharing a letter are significantly different. Table S2. Environmental fitness of environmental parameters correlated with DCA ordinations of Generalists, Common taxa, and Specialists. Table S3. Module hubs and connectors in Total network and their topological characteristics and taxonomical features. Figure S1. The distribution of the niche breadthvalues of the ASVs. A B-value of >78 was chosen as a criterion for generalists as this value lies within the outlier area of the B-value distribution, while ASVs with B-values of <22 were regarded as specialists. Figure S2 Shannon diversity index of the nine different sampling sites in Salluit, Nunavik. Figure S3.The relative abundance of Generalist, Common taxa, and Specialist modules in the total network module.Composition of major modules. Figure S4. Spearman's rank correlation matrix between the variables including the distribution patterns of individual modules and environmental parameters. The colors of the scale bar represent Spearman's correlation coefficient [file 40793_2023_498_MOESM1_ESM.docx]

**Supplementary materials**

Table S1. Results of one-way ANOVA and post-hoc Tukey’s HSD testing among-elevation variation in environmental and biotic variables. For the Tukey HSD column, elevation not sharing a letter are significantly different (p<0.05).

| **Variables** | **F** | ***df*** | ***p*** | **Tukey HSD (Mean)** |
| --- | --- | --- | --- | --- |
| Respiration | 2.73 | 2 | 0.067 |  |
| Temperature | 127.60 | 2 | <0.001 | High (17.14)^a^ > Middle (13.46)^b^ > Low(10.23)^c^ |
| Moisture | 108.20 | 2 | <0.001 | High (17.87)^c^ < Middle (25.80)^b^ < Low (58.79)^a^ |
| Dry/Wet Weight Ratio | 103.0 | 2 | <0.001 | High (0.89)^a^ > Middle (0.79)^b^ > Low (0.60)^c^ |
| C/N Ratio | 8.57 | 2 | <0.001 | High (12.88)^b^ , Middle (12.42)^b^ < Low (14.05)^a^ |
| pH | 0.062 | 2 | 0.94 |  |
| *Coverage* |  |  |  |  |
| Rock | 22.63 | 2 | <0.001 | High (15.43)^a^ > Middle (9.60)^b^ > Low (4.72)^c^ |
| Cryptogram | 12.69 | 2 | <0.001 | High (40.13)^a^ > Middle (29.77)^b^ , Low (24.95)^b^ |
| Sediment | 30.59 | 2 | <0.001 | High (33.25)^a^ > Middle (20.63)^b^ > Low(8.24)^c^ |
| Vascular Plant | 186.00 | 2 | <0.001 | High (18.32)^c^ < Middle(45.80)^b^ < Low (70.47)^a^ |

Table S2. Environmental fitness of environmental parameters correlated with DCA ordinations of Generalists, Common taxa, and Specialists (Fig. 1).

|  | Generalists | | | | Common taxa | | | | Specialists | | | | |
| --- | --- | --- | --- | --- | --- | --- | --- | --- | --- | --- | --- | --- | --- |
|  | DCA1 | DCA2 | *r*^2^ | *P* | DCA1 | DCA2 | *r*^2^ | *P* | DCA1 | DCA2 | *r*^2^ | *P* |  |
| Respiration | 0.83 | -0.55 | 0.01 | 0.532 | 0.82 | 0.58 | 0 | 0.807 | -0.87 | 0.49 | 0 | 0.95 |  |
| Temperature | -0.94 | -0.35 | 0.11 | **0.001** | -1.00 | 0.01 | 0.10 | **0.001** | -0.87 | 0.49 | 0.14 | **0.001** |  |
| Moisture | 0.84 | 0.54 | 0.05 | 0.003 | 0.96 | -0.28 | 0.06 | **0.001** | 0.88 | -0.47 | 0.10 | **0.001** |  |
| C/N Ratio | -0.98 | -0.22 | 0.10 | **0.001** | -0.94 | 0.34 | 0.17 | **0.001** | -0.98 | 0.21 | 0.18 | **0.001** |  |
| pH | 0.94 | -0.34 | 0.05 | 0.003 | 0.89 | 0.46 | 0.07 | **0.001** | 0.84 | 0.54 | 0.11 | **0.001** |  |
| Ec | 0.98 | 0.20 | 0.06 | 0.002 | 0.98 | 0.18 | 0.07 | **0.001** | 0.98 | -0.20 | 0.09 | **0.001** |  |
| Elevation | -0.93 | -0.38 | 0.26 | **0.001** | -0.99 | 0.16 | 0.25 | **0.001** | -0.93 | 0.38 | 0.29 | **0.001** |  |
| *Coverage* |  |  |  |  |  |  |  |  |  |  |  |  |  |
| Rock | -0.97 | -0.23 | 0.03 | 0.036 | -0.97 | -0.23 | 0.03 | 0.064 | -0.90 | -0.43 | 0.02 | 0.164 |  |
| Cryptogram | 0.39 | -0.92 | 0.01 | 0.489 | 0.56 | 0.83 | 0 | 0.584 | 0.16 | -0.99 | 0.01 | 0.625 |  |
| Sediment | -0.71 | -0.71 | 0.02 | 0.148 | -0.78 | 0.62 | 0.01 | 0.303 | -0.57 | 0.82 | 0.03 | 0.042 |  |
| Vascular Plant | 0.74 | 0.67 | 0.09 | **0.001** | 0.80 | -0.60 | 0.07 | **0.001** | 0.88 | -0.47 | 0.08 | **0.001** |  |

Table S3. Module hubs and connectors in Total network and their topological characteristics and taxonomical features

| ASV.no | Role | Niche breadth | C.score | Z.score | Node degree | Betweenness | Assigned module ID | Phylum | Class | Order |
| --- | --- | --- | --- | --- | --- | --- | --- | --- | --- | --- |
| ASV00009 | Connector | Generalist | 0.7 | -0.8 | 3 | 807.029 | T_Module IV | Acidobacteriota | Acidobacteriae | Acidobacteriae Subgroup 2 |
| ASV00072 | Connector | Generalist | 0.7 | -0.7 | 6 | 1108.236 | T_Module IV | Actinobacteriota | Thermoleophilia | Gaiellales |
| ASV00225 | Connector | Generalist | 0.6 | 0.7 | 22 | 10581.9 | T_Module IV | Proteobacteria | Alphaproteobacteria | Rhizobiales |
| ASV00383 | Connector | Common taxa | 0.7 | -0.7 | 6 | 9569.996 | T_Module V | Chloroflexi | Ktedonobacteria | Ktedonobacterales |
| ASV00800 | Connector | Common taxa | 0.7 | -0.5 | 7 | 1271.356 | T_Module III | Chloroflexi | Ktedonobacteria | Ktedonobacterales |
| ASV00559 | Connector | Common taxa | 0.6 | -0.6 | 4 | 1437.69 | T_Module III | Acidobacteriota | Vicinamibacteria | Vicinamibacterales |
| ASV00147 | Module hub | Generalist | 0.1 | 2.6 | 109 | 11690.94 | T_Module I | Acidobacteriota | Holophagae | Holophagae Subgroup 7 |
| ASV00038 | Module hub | Generalist | 0.5 | 3.7 | 62 | 25285.63 | T_Module IV | Proteobacteria | Alphaproteobacteria | Rhizobiales |
| ASV00008 | Module hub | Generalist | 0.1 | 3.0 | 38 | 45877.65 | T_Module V | Proteobacteria | Gammaproteobacteria | WD260 |
| ASV00110 | Module hub | Common taxa | 0.2 | 4.1 | 52 | 28366.95 | T_Module V | Proteobacteria | Alphaproteobacteria | Acetobacterales |
| ASV00130 | Module hub | Common taxa | 0.4 | 3.4 | 54 | 7421.026 | T_Module V | Acidobacteriota | Acidobacteriae | Acidobacteriae Subgroup 2 |
| ASV00142 | Module hub | Common taxa | 0.1 | 2.5 | 109 | 8929.401 | T_Module II | Acidobacteriota | Acidobacteriae | Acidobacteriales |
| ASV00255 | Module hub | Common taxa | 0.4 | 3.4 | 59 | 8637.462 | T_Module VI | Acidobacteriota | Acidobacteriae | Bryobacterales |
| ASV00344 | Module hub | Common taxa | 0.1 | 3.0 | 121 | 14192.44 | T_Module I | Acidobacteriota | Acidobacteriae | Bryobacterales |
| ASV00191 | Module hub | Common taxa | 0.1 | 2.6 | 107 | 14569.8 | T_Module I | Proteobacteria | Gammaproteobacteria | Burkholderiales |
| ASV00928 | Module hub | Common taxa | 0.1 | 2.9 | 114 | 7761.666 | T_Module I | Proteobacteria | Alphaproteobacteria | Caulobacterales |
| ASV00259 | Module hub | Common taxa | 0.0 | 2.8 | 37 | 9506.318 | T_Module III | Bacteroidota | Bacteroidia | Chitinophagales |
| ASV00751 | Module hub | Common taxa | 0.0 | 2.8 | 37 | 3640.511 | T_Module III | Bacteroidota | Bacteroidia | Chitinophagales |
| ASV00213 | Module hub | Common taxa | 0.5 | 3.0 | 80 | 13643.65 | T_Module VI | Verrucomicrobiota | Verrucomicrobiae | Chthoniobacterales |
| ASV00240 | Module hub | Common taxa | 0.5 | 4.6 | 54 | 35495.63 | T_Module IV | Verrucomicrobiota | Verrucomicrobiae | Chthoniobacterales |
| ASV00551 | Module hub | Common taxa | 0.0 | 2.8 | 37 | 13788.13 | T_Module III | Verrucomicrobiota | Verrucomicrobiae | Chthoniobacterales |
| ASV00115 | Module hub | Common taxa | 0.4 | 4.4 | 63 | 18667.25 | T_Module V | Actinobacteriota | Actinobacteria | Frankiales |
| ASV00153 | Module hub | Common taxa | 0.0 | 3.0 | 118 | 7856.094 | T_Module II | Actinobacteriota | Actinobacteria | Frankiales |
| ASV00465 | Module hub | Common taxa | 0.1 | 3.9 | 50 | 11495.19 | T_Module III | Proteobacteria | Gammaproteobacteria | Gammaproteobacteria Incertae Sedis |
| ASV00601 | Module hub | Common taxa | 0.0 | 3.3 | 124 | 14771.11 | T_Module I | Gemmatimonadota | Gemmatimonadetes | Gemmatimonadales |
| ASV00632 | Module hub | Common taxa | 0.4 | 4.5 | 74 | 40610.88 | T_Module III | Proteobacteria | Gammaproteobacteria | JG36-TzT-191 |
| ASV00148 | Module hub | Common taxa | 0.1 | 2.6 | 25 | 17313.46 | T_Module IV | Chloroflexi | Ktedonobacteria | Ktedonobacterales |
| ASV00216 | Module hub | Common taxa | 0.0 | 2.6 | 23 | 3420.31 | T_Module IV | Chloroflexi | Ktedonobacteria | Ktedonobacterales |
| ASV00234 | Module hub | Common taxa | 0.0 | 3.1 | 120 | 6411.158 | T_Module II | Chloroflexi | Ktedonobacteria | Ktedonobacterales |
| ASV00256 | Module hub | Common taxa | 0.0 | 2.7 | 108 | 3552.733 | T_Module II | Chloroflexi | Ktedonobacteria | Ktedonobacterales |
| ASV00070 | Module hub | Common taxa | 0.0 | 4.5 | 159 | 29082.51 | T_Module I | Actinobacteriota | Acidimicrobiia | Microtrichales |
| ASV00041 | Module hub | Common taxa | 0.5 | 2.9 | 62 | 64912.5 | T_Module V | Proteobacteria | Alphaproteobacteria | Rhizobiales |
| ASV00251 | Module hub | Common taxa | 0.2 | 3.7 | 47 | 14075.61 | T_Module V | Proteobacteria | Alphaproteobacteria | Rhizobiales |
| ASV00287 | Module hub | Common taxa | 0.5 | 2.7 | 41 | 13295.99 | T_Module IV | Proteobacteria | Alphaproteobacteria | Rhizobiales |
| ASV00292 | Module hub | Common taxa | 0.0 | 3.9 | 141 | 17162.91 | T_Module I | Proteobacteria | Alphaproteobacteria | Rhizobiales |
| ASV00326 | Module hub | Common taxa | 0.1 | 2.7 | 39 | 26386.5 | T_Module III | Proteobacteria | Alphaproteobacteria | Rhizobiales |
| ASV00367 | Module hub | Common taxa | 0.0 | 2.6 | 107 | 5554.266 | T_Module II | Proteobacteria | Alphaproteobacteria | Rhizobiales |
| ASV00626 | Module hub | Common taxa | 0.1 | 2.5 | 103 | 9771.259 | T_Module I | Proteobacteria | Alphaproteobacteria | Rhizobiales |
| ASV00829 | Module hub | Common taxa | 0.4 | 2.6 | 30 | 7938.818 | T_Module IV | Proteobacteria | Alphaproteobacteria | Rhizobiales |
| ASV00166 | Module hub | Common taxa | 0.2 | 3.5 | 48 | 132101.8 | T_Module III | Planctomycetota | Phycisphaerae | Tepidisphaerales |
| ASV01652 | Module hub | Common taxa | 0.0 | 3.9 | 48 | 29742.9 | T_Module III | Planctomycetota | Phycisphaerae | Tepidisphaerales |
| ASV01918 | Module hub | Common taxa | 0.1 | 3.0 | 42 | 6017.626 | T_Module III | Planctomycetota | Phycisphaerae | Tepidisphaerales |
| ASV00616 | Module hub | Common taxa | 0.3 | 2.9 | 31 | 8710.969 | T_Module IV | Chloroflexi | Gitt-GS-136 | unclassified Gitt-GS-136 |
| ASV00218 | Module hub | Common taxa | 0.5 | 4.0 | 76 | 24719.29 | T_Module VI | Chloroflexi | TK10 | unclassified TK10 |
| ASV00446 | Module hub | Common taxa | 0.2 | 5.4 | 72 | 26855.36 | T_Module III | Acidobacteriota | Vicinamibacteria | Vicinamibacterales |
| ASV00138 | Module hub | Specialist | 0.5 | 2.6 | 60 | 8631.699 | T_Module VI | Chloroflexi | Ktedonobacteria | Ktedonobacterales |
| ASV00922 | Module hub | Specialist | 0.1 | 2.6 | 24 | 2422.926 | T_Module IV | Chloroflexi | Ktedonobacteria | Ktedonobacterales |
| ASV00903 | Module hub | Specialist | 0.1 | 3.2 | 29 | 5285.609 | T_Module IV | Chloroflexi | AD3 | unclassified AD3 |


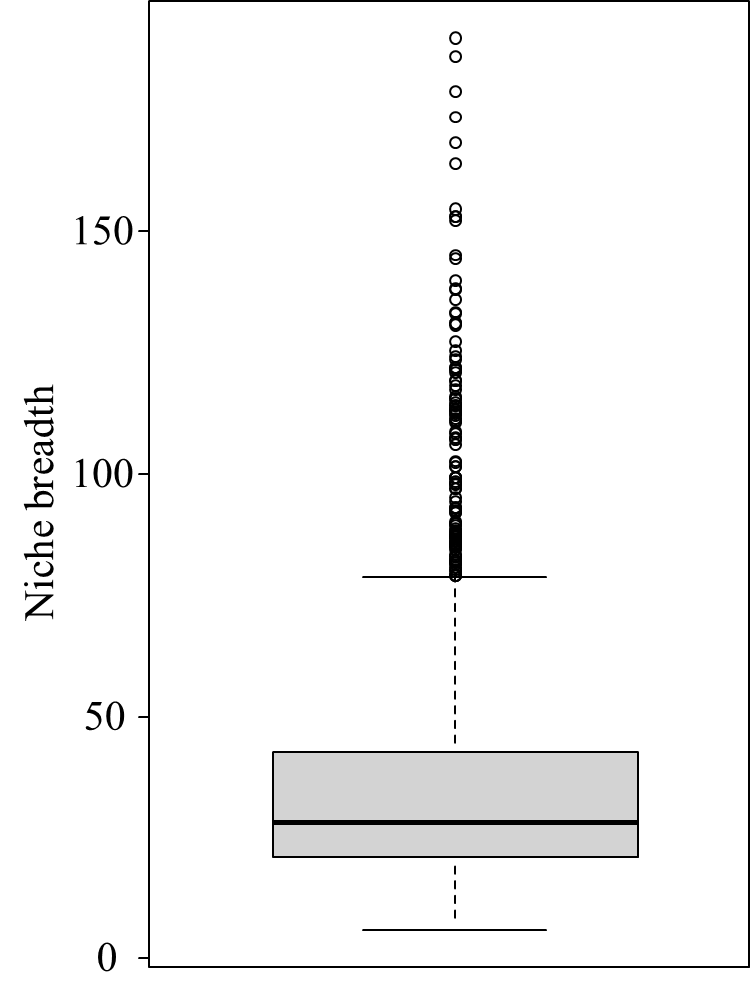


**Figure S1** The distribution of the niche breadth (B) values of the ASVs. A B-value of >78 was chosen as a criterion for generalists as this value lies within the outlier area of the B-value distribution, while ASVs with B-values of <22 were regarded as specialists (lower quartile).


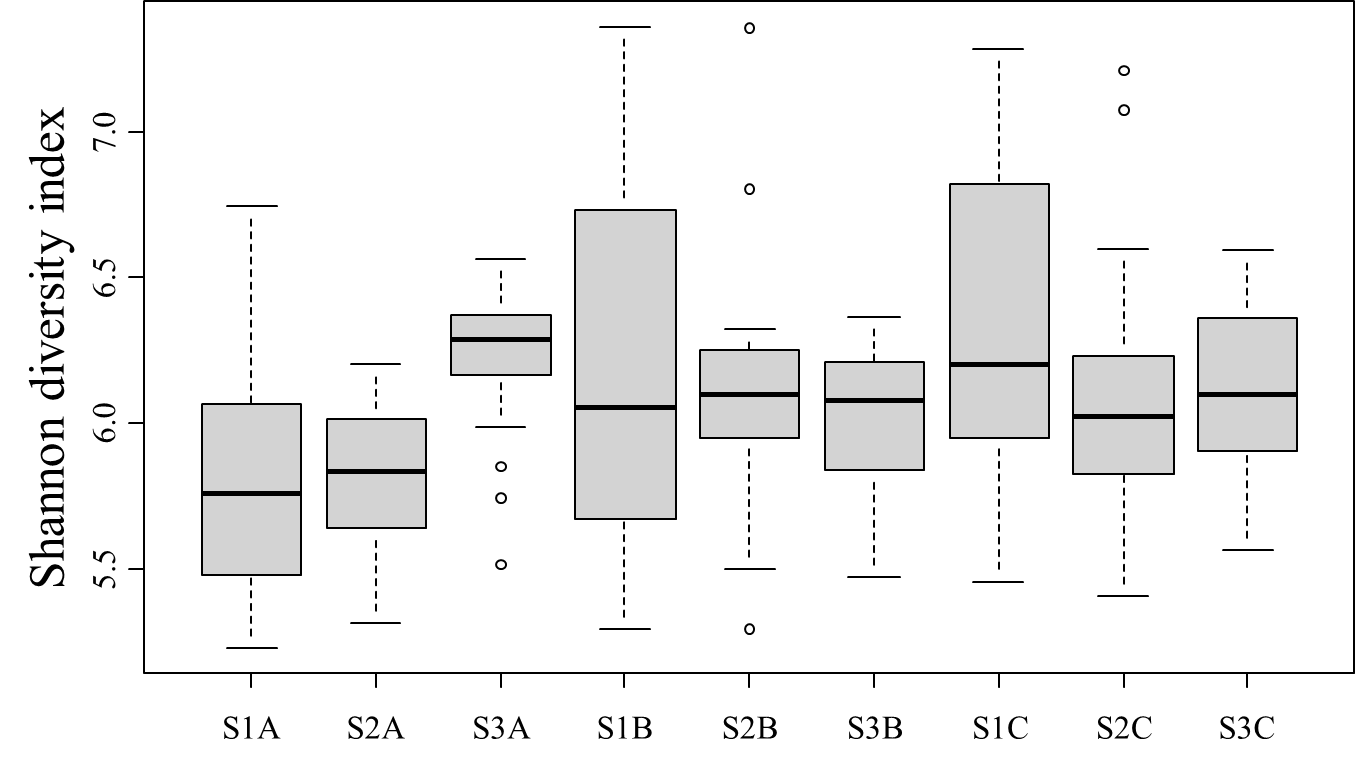


**Figure S2** Shannon diversity index of the nine different sampling sites in Salluit, Nunavik.


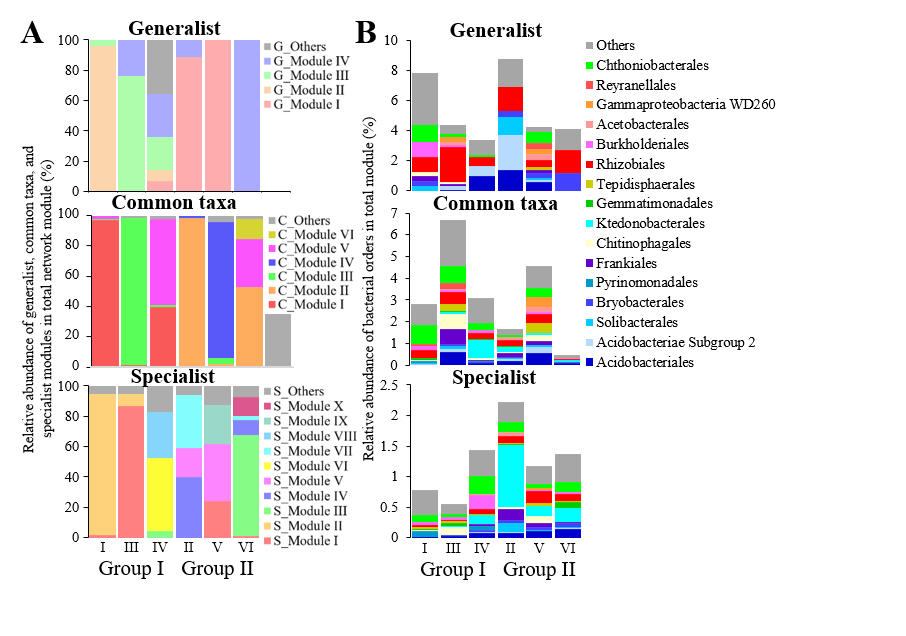


**Figure S3** (A) The relative abundance of Generalist, Common taxa, and Specialist modules in the total network module. (B) Composition of major modules.


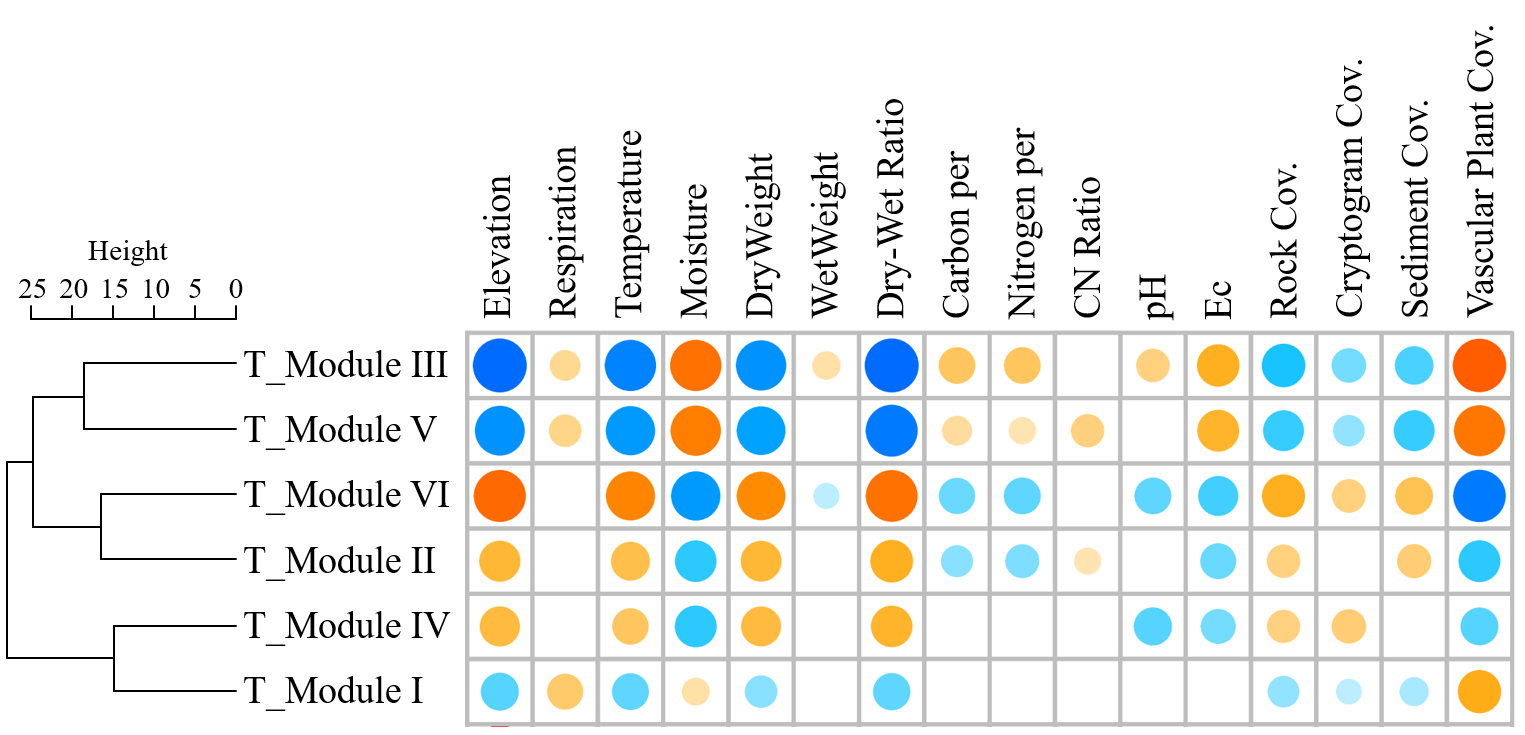


**Figure S4.** Spearman's rank correlation matrix between the variables including the distribution patterns of individual modules and environmental parameters. The colors of the scale bar represent Spearman's correlation coefficient (*ρ*).
